# Epigenetic Regulation Through Histone Deacetylation: Implications and Therapeutic Potential in Hepatocellular Carcinoma

**DOI:** 10.3390/cells14171337

**Published:** 2025-08-29

**Authors:** Khulah Sadia, Annalisa Castagna, Silvia Udali, Francesca Ambrosani, Patrizia Pattini, Ruggero Beri, Giuseppe Argentino, Maria Masutti, Sara Moruzzi, Simonetta Friso

**Affiliations:** Department of Medicine, Section of Internal Medicine, University of Verona, Piazzale L.A. Scuro, 37134 Verona, Italy; khulah.sadia@univr.it (K.S.); annalisa.castagna@univr.it (A.C.); silvia.udali@univr.it (S.U.); francesca.ambrosani@univr.it (F.A.); patrizia.pattini@univr.it (P.P.); ruggero.beri@univr.it (R.B.); giuseppe.argentino@univr.it (G.A.); maria.masutti@studenti.univr.it (M.M.); sara.moruzzi@aovr.veneto.it (S.M.)

**Keywords:** histone deacetylases, HDACs, epigenetics, hepatocellular carcinoma, synthetic HDAC inhibitors, natural HDAC inhibitors, combination therapy

## Abstract

Hepatocellular carcinoma (HCC) is a leading cause of global cancer-related mortality worldwide. Increasing evidence indicates that epigenetic mechanisms, which are potentially reversible and modifiable by environmental and nutritional factors, play a key role in hepatocarcinogenesis. Histone deacetylases (HDACs) are fundamental epigenetic modulators that regulate chromatin dynamics and ultimately gene transcription with important pathophysiological implications and promising therapeutic perspectives. The role of HDACs is gaining interest for the understanding of HCC development mechanisms and for the potential therapeutic implications of their natural and synthetic inhibitors. This review provides an overview on HDACs classification and their peculiar expression patterns in HCC, with a focus on zinc-dependent histone deacetylases (HDACs). HDAC inhibitors (HDACis), both synthetic and natural-derived compounds, are also discussed for their emerging effects in optimizing the anticancer efficacy of the current therapeutic strategies. Novel dietary-derived and bioactive compounds-based interventions are discussed in the context of HCC management as promising nutri-epigenetic avenues. Targeting HDACs bears a significant therapeutic potential for HCC management while further confirmatory clinical investigation is warranted.

## 1. Introduction

### 1.1. Epigenetic Landscape

Epigenetics refers to hereditary changes in gene expression that occur without alterations in the underlying DNA sequence [1,2]. Epigenetic modifications involve heritable changes that allows specific DNA regions to interact or repel regulatory proteins, such as transcription factors or transcription enhancers/silencers, thereby causing the activation or repression of the expression of specific genes in different cell types [1]. Broadly studied epigenetic mechanisms include DNA methylation, histone modifications, and non-coding RNAs (ncRNAs). The main ncRNAs involved in gene regulation and transcription include microRNA, small interfering RNA, long ncRNA, and piwi-interacting RNA [3]. DNA methylation is considered one of the major epigenetic modifications of DNA and was extensively studied in different pathologies [4,5,6,7]. The role of epigenetic features of DNA are especially fascinating because of their potential reversibility by environmental and nutritional factors [8,9,10]. DNA methylation patterns play a dual role in cancer, encompassing both gene-specific and genomic alterations [4,6]. Promoter hypermethylation is a hallmark of transcriptional repression and is often observed in the context of silencing tumor suppressor genes (TSGs) that are critical for cell cycle regulation. On the contrary, global genomic hypomethylation, occurring specifically in repetitive elements, impacts genomic stability and peculiar oncogene activation [11,12]. Recent studies have further emphasized the importance of this methylation pattern in maintaining cellular homeostasis [13,14], liver plasticity [15], iron metabolism and epigenetic regulation in hepatocellular carcinoma (HCC) [4,5,16,17]. The interplay between histone acetylation and deacetylation patterns play a key role in the regulation of the transcriptional machinery in different carcinoma including liver cancer [18,19], colorectal cancer [20] and pancreatic cancer [21]. These epigenetic modifications have been significantly implicated in the development and progression of malignancies through several molecular pathways [22,23,24]. Such alterations are recognized as significant contributors to disease heterogeneity and drug resistance. The epigenetic landscape is dynamically regulated, enabling changes in cell fate through transcriptional reprogramming. Epigenetic modifications are reversible, thus presenting an opportunity for anticancer therapies [25,26] that can target, for example, different histone modifications [27]. This review aims to present an up-to-date understanding of post-translational histone modifications, particularly histone acetylation/deacetylation, in the context of HCC, and to explore their potential implications for the development of targeted therapeutic strategies. Moreover, by considering the key role of nutritional factors in the regulation of epigenetic mechanisms such as those related to HDACs function, this review addresses also the current knowledge on the interaction between HDACs inhibitors and nutrients, highlighting their contribution in current therapeutic strategies and opening to precision therapeutic targeting.

### 1.2. Post-Translational Histone Modifications

Histone proteins are key players in epigenetics. Histones are bound to DNA in the nucleus and belong to a family of basic proteins with five main components, i.e., H1, H2A, H2B, H3, and H4. Nucleosome is the fundamental unit of chromatin which comprises approximately 147bp DNA wrapped around a histone octamer composed of two copies of H3, H4, H2A and H2B proteins. These nucleosomal histones can be chemically modified [28,29]. Chromatin structure and function is regulated through the action of histone post-translational modifications (PTMs). Almost half a century has passed since Vincent Allfrey first explained the presence of histone acetylation and methylation [30] while their functional significance remained uncertain for many years. Although fundamental breakthroughs have been made in understanding the function of histone PTMs through the identification of the protein machineries that encompass add (write), eliminate (erase) and bind (read) modifications, there are still open questions on how histone PTMs act in chromatin regulation. Histone modifications have been recognized as a pivotal mechanism governing gene transcription that substantially effects cancer induction, progression and metastasis [31]. Extensively studied PTMs include histone lysine acetylation and deacetylation [32,33], lysine and arginine methylation [34,35,36], arginine citrullination, lysine ubiquitination and lysine sumoylation [37,38,39,40,41]. Histone acetylation and deacetylation play a critical and prominent role in epigenetic modulation and regulation of gene expression. Histone acetyltransferases (HATs) and histone deacetylases (HDACs) are two opposing classes of enzymes that play crucial functions in the regulation of transcription either by changing chromatin structure or by modulating the activity of specific transcription factors [42].

## 2. Dynamics of Histone Acetylation/Deacetylation

**Figure 1:**
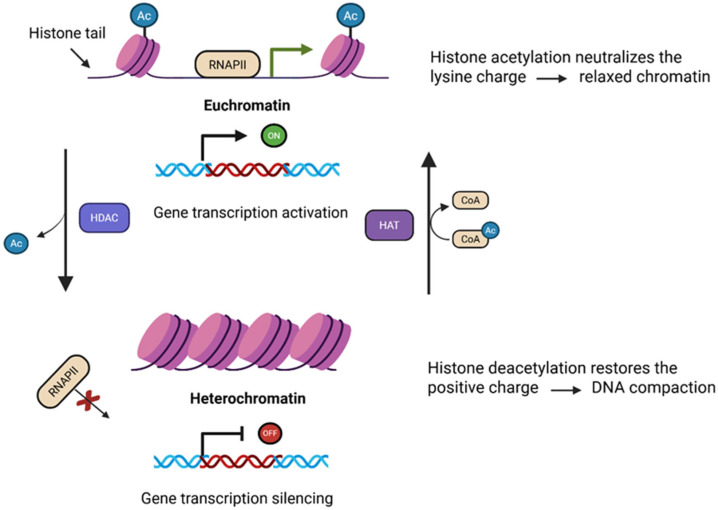
Overview of the HAT-HDAC mediated epigenetic transcriptional regulation. HDAC removes the acetyl groups (Ac) from histone tail forming closed chromatin structure (Heterochromatin) thereby reducing transcriptional activity of RNA polymerase II, while HAT catalyzes the transfer of acetyl groups from acetyl-CoA at the N-terminal histone tail resulting in an open chromatin state (Euchromatin) facilitating gene transcription. Created with BioRender.com.

The chemical properties of histones, which carry a positive charge, promote their strong interaction with the negatively charged DNA, leading to tight binding between the two. Histone acetylation occurs mostly at the N-terminal lysine residues of histones H3 and H4 and leads to transcriptional active chromatin, i.e., open chromatin or euchromatin. Histone acetyltransferases (HATs) transfer the acetyl group of acetyl coenzyme A to histone proteins within the N-terminal tail protruding from the histone core at specific lysine residues, thereby neutralizing the positive charge of histones ultimately weakening the interaction between DNA and histones and relaxing the structure of the nucleosome (Figure 1) and it leads to transcriptional active chromatin, i.e., open chromatin or euchromatin. HATs are classified into two general classes based on their cellular origin and function. Cytoplasmic HATs include B-type HATs that likely catalyze acetylation events associated with the transfer of newly synthesized histones from the cytoplasm to the nucleus. On the contrary, nuclear HATs are A-type HATs catalyzing transcription-related acetylation events [43,44]. In contrast, the action of Histone deacetylases (HDACs), which remove acetyl groups from histone tails, allows histones to tightly bind to negatively charged DNA and repressing gene transcription, leading to a more compact chromatin structure associated with transcriptional repression, i.e., heterochromatin [45,46] (Figure 1). HDACs play a critical role in epigenetic regulation through precise molecular interactions and catalytic mechanisms. These enzymes mediate chromatin remodeling orchestrating intricate cellular processes through sophisticated protein complex formations, including interactions with co-repressors like Sin3A and NuRD complexes [47,48], modulating gene expression across different cellular contexts. HDACs have recently been shown to modify a variety of other proteins that are involved in different cellular processes [26,49,50,51]. The enzymatic mechanism of HDACs involves a nuanced zinc-dependent hydrolysis process, characterized by substrate-specific recognition, conformational changes, and differential regulation across four distinct classes. Each HDAC class exhibits unique structural characteristics and functional specificity, ranging from ubiquitously expressed nuclear Class I HDACs to specialized metabolic sensing Class III sirtuins, ultimately contributing to complex epigenetic networks that regulate transcription, cell cycle progression, and cellular differentiation through precise molecular interactions and catalytic activities [52,53]. Disruption of the balance between HATs and HDACs activities can result in aberrant expression of specific genes. Inhibition of HAT activity may delay the proper timing of target gene expression, whereas blocking HDAC activity could result in prolonged or continuous expression of the same gene. The imbalance between HATs and HDACs has been described having a role in the silencing of TSGs and in cancer induction [22,54,55]. Therefore, tightly regulating the functions of HATs and HDACs is essential to ensure accurate and timely expression of genes involved in signal transduction, as well as in processes such as cell proliferation and death.

## 3. HDACs Classification

**Figure 2:**
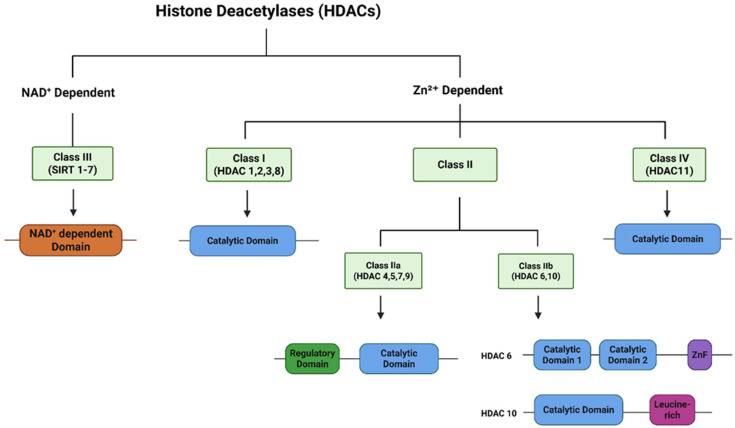
Classification and domain architecture of the HDACs Family. HDACs can be classified as NAD+ dependent (Class III) and Zinc dependent (Class I, II and IV). ZnF, ubiquitin binding zinc finger domain; Leucine-rich, Leucine-rich region.

The known histone deacetylase homologs in mammals are eighteen. Histone deacetylases are divided into four classes: class I (HDACs 1, 2, 3, and 8), class II (HDACs 4, 5, 6, 7, 9, and 10), class III (sirtuins, SIRT1-7), and class IV (HDAC11). Figure 2 explains the classification and domain architecture of all HDACs. The first human HDAC was discovered in 1998 and was named HDAC1 [56], followed by HDAC2 [57] and HDAC3 [58]. In humans, the HDAC family characterization was last updated in 2002 with the discovery of HDAC11 [59]. All classes of HDACs are numbered according to their chronological order of discovery and are grouped according to their homology to yeast orthologues. Histone deacetylases from 1 to 11 are zinc dependent while Class III sirtuins require NAD+ as a cofactor [60]. HDAC1 is considered the prototype of the HDAC family. Class I and II HDACs are widely studied and reported to be associated with cancer pathogenesis, thus, this literature review is focused on these zinc-dependent histone deacetylases.

### 3.1. Class I HDACs

Class I mammalian HDACs include HDAC 1, 2, 3, and 8. These HDACs are composed of an entirely conserved deacetylase domain and possess sequence similarity to the yeast HDAC, reduced potassium dependency protein (Rpd3), which is responsible for the deacetylation of lysine residues on the N-terminal part of the core histones in yeast [61]. Class I HDACs are primarily located in the nucleus, where they present strong deacetylase activity toward histones. They mainly operate in groups where multiple HDACs like HDAC1 and HDAC2 exist together in at least three different multiprotein complexes: nucleosome remodeling and deacetylase complex (NuRD), corepressor of REST (CoREST) and transcriptional regulatory protein Sin3A. The non-conserved C-terminal region of HDAC3 is required for both deacetylase activity and transcriptional repression. HDAC3 activity depends on SMRT (silencing mediator for retinoic acid and thyroid hormone receptors) and N-CoR (nuclear receptor co-repressor) which contain deacetylase activating domains [62]. The last member of HDAC class I is HDAC8 with a structure simpler than other class I HDACs, consisting mainly of a catalytic domain with a central nuclear localization signal (NLS) which functions alone [63,64,65]. Nevertheless, its regulatory complex and protein interactions remain unknown. In addition to histones, Class I HDACs deacetylate several non-histone proteins because of their predominant nuclear localization. Table 1 shows the histone and non-histone substrates as well as the cellular localization of all HDACs.

### 3.2. Class II HDACs

Class II HDACs have sequence homology with yeast HDAC1, the putative catalytic subunit of histone deacetylase complex in yeast and exhibit a conserved deacetylase domain at their C-terminus [66]. Generally, class II is subdivided into two sub-classes: Class IIa and Class IIb, based on sequence analysis. HDAC class IIa involves HDAC4, 5, 7, and 9 which possess a unique adapter domain in the N-terminus that forms a binding site for the DNA-binding transcription factor and myocyte enhancer factor 2 (MEF2). Nucleocytoplasmic shuttling is the distinctive characteristic of class II HDACs which demonstrates cell type specificity and signal dependence. Class IIa HDACs also form a large complex with the SMRT/N-CoR-HDAC3. Intrinsically, this class has low enzymatic activity. Class I HDACs have a conserved tyrosine residue in the catalytic site whereas in class IIa HDACs this is substituted with histidine [67,68]. Thus, class IIa HDACs may function as deacetylases with low enzymatic activity or may possess specific targets that have yet to be identified. Ectopic expression studies of HDAC5 and HDAC7 revealed novel nuclear structures called matrix associated deacetylase (MAD) bodies whose formation is determined by deacetylase activity. These MAD bodies contain several proteins and also components of the NuRD/Mi2/NRD and Sin3/HDAC complexes [69]. HDAC9 catalytic domain is located on the N-terminus, and it also possesses a conserved deacetylase domain. When HDAC9 is recruited to a promotor, this results in the repression of gene activity through deacetylation of histones. Additionally, HDAC9 also interacts with MEF2 [70]. There are three reported HDAC9 splice variants with different protein functions distinguished as HDAC9a, HDAC9b, and HDRP/HDAC9c [71].

Class IIb HDACs comprise HDAC6 and HDAC10. HDAC6 contains two deacetylase domains and a C-terminal zinc finger ubiquitin-binding domain commonly known as HDAC6, USP3-, Brap2-related zinc finger motif (HUB) [72]. It comprises distinct structural domains such as nuclear localization signal region (NLS), tandem deacetylation catalytic regions (DD1, DD2), serine–glutamate-containing tetradecapeptide repeat region (SE14), leucine-rich nuclear export signal regions (NES1, NES2) and ubiquitin-binding zinc finger structure (ZnF-UBP). Despite containing a nuclear localization signal, HDAC6 cytoplasmic localization is primarily governed by NES and SE14, facilitating its translocation and anchoring in the cytoplasm [73,74]. Structurally, HDAC10 features an N-terminal catalytic domain, a nuclear export signal (NES), and a possible second catalytic domain at its C-terminus. It also contains two putative Rb binding domains. HDAC10 also operates as a transcriptional repressor, capable of shuttling between the nucleus and cytoplasm. Analysis of protein sequence identity shows that HDAC10 is most closely related (37% overall similarity) to HDAC6 [75]. Although HDAC10 demonstrates autonomous deacetylase activity when recombinantly expressed, its capability to interact with multiple HDACs (1, 2, 3, 4, 5, and 7) but not with HDAC6 proposes the idea that it might primarily function as a recruiting protein.

### 3.3. Class III HDACs

The class III HDACs share homology with the yeast silent information regulator 2 (Sir2), a protein essential for transcriptional silencing, and are evolutionarily conserved across a broad range of species from bacteria to humans. A defining feature of class III HDACs is the presence of a deoxyhypusine synthase-like domain capable of binding NAD and FAD cofactors, distinguishing them from other HDAC classes. In humans, seven Sir2 homologs, collectively known as sirtuins (SIRT1-7), have been identified. Notably, sirtuins possess additional enzymatic functions, including mono-ADP-ribosyltransferase activity, beyond their histone deacetylase role. Sirtuins share 22–50% overall amino acid sequence similarity and 27–88% resemblance in their conserved catalytic domains [53,60]. These proteins are localized in various cellular compartments, including the nucleus (SIRT1, SIRT2, SIRT3, SIRT6, and SIRT7), the cytoplasm (SIRT1 and SIRT2), and the mitochondria (SIRT3, SIRT4, and SIRT5) [76].

### 3.4. Class IV HDACs

Class IV histone deacetylase includes only HDAC11, which is homologous to yeast Hos3 and shares a catalytic domain with both HDACs of class I and class II. HDAC11 exhibits sequence homology to Rpd3 and HDAC1 proteins [61]. The highly conserved dynamic residues of HDAC11 in the catalytic core region also share similarity with both class I and II HDACs. HDAC11 is the shortest isoform and is primarily composed of the core catalytic domain that exhibits exclusive deacetylase activity [77]. In vivo, HDAC11 can also form complexes with HDAC6, but the 3D structure is not yet available. The expression of HDAC11 can be regulated by temperature [78].

## 4. HDACs Expression Patterns in Hepatocellular Carcinoma

Liver cancer is the sixth most diagnosed cancer worldwide and the third leading cause of cancer-related deaths with over 750,000 deaths annually. The incidence and mortality rate of liver cancer are two to three times higher in men than women [79].

Hepatocellular carcinoma is the most common type of primary liver cancer comprising the 75–85% of liver cancer cases. The age of HCC occurrence varies globally but the median age considered for its onset is >60 years [80]. The primary risk factors for HCC include HBV or HCV chronic infection, aflatoxin exposure, heavy alcohol consumption, excess body weight, type 2 diabetes, and smoking [79]. Although hepatocellular carcinoma predominantly develops in cirrhotic livers, a notable proportion of cases (approximately 15–20%) arises in non-cirrhotic individuals, particularly in the context of metabolic dysfunction-associated steatotic liver disease [81]. The pathogenesis of HCC is a complex, multifactorial process influenced by genetic alterations, epigenetic modifications, and environmental exposures that collectively contribute to malignant transformation. The treatment options range from locoregional treatments, including ablation, and surgical resection, to liver transplantation and systemic therapy for more advanced stages cases [82].

**Figure 3:**
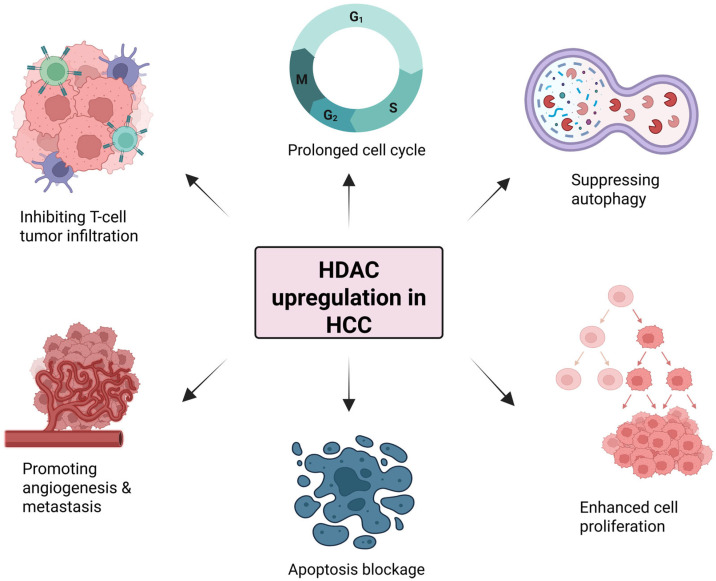
Impact of HDAC upregulation in hepatocellular carcinoma (HCC). HDAC overexpression is linked with cancer cell proliferation, promotion of angiogenesis, and metastases as well as inhibition of T-cell tumor infiltration and cancer cell autophagy and apoptosis. Created with BioRender.com.

Several studies have reported that histone deacetylases are linked with tumor progression in different cancer types including HCC, where HDACs are usually highly overexpressed [83,84]. Overall, HDACs upregulation, in particular that of zinc-dependent HDACs, is associated with cancer cell proliferation, promotion of angiogenesis, and metastases and inhibition of T-cell tumor infiltration (Figure 3). HDAC1 is the most widely studied member of the HDAC family, demonstrating anomalous high expression in HCC tissues and cell lines [85,86,87]. Likewise, Buurman and colleagues have reported upregulated expression of *HDAC1-3* in both HCC patients and cell lines. They demonstrated that the HDAC inhibition in HCC cells through Trichostatin A induces apoptosis and decreases proliferation, partly by upregulating miR-449 leading to reduced ERK1/2 signaling [88]. Similarly, elevated HDAC1 and HDAC2 expression has been associated with increased mortality in HCC patients and in vitro studies showed that simultaneous knockdown of HDAC1/2 resulted in reduced cell proliferation, colony formation, and survival in HCC cell lines [89]. Lachenmayer and colleagues reported the significant upregulated expression of *HDAC2*, *HDAC4*, and *HDAC11* in a cohort of liver cancer patients, whereas *HDAC3* and *HDAC5* were found to be associated with copy number gains in human HCC [90]. Another study reported the elevated expression of HDAC5 in HCC and its downregulation in HCC cell lines contributed to apoptosis induction and cell cycle arrest [91]. Furthermore, HDAC5 has been shown to facilitate HCC metastasis under hypoxia by repressing HIPK2, stabilizing hypoxia-inducible factor 1α (HIF1α), and promoting epithelial–mesenchymal transition (EMT) and angiogenesis [92]. LukS-PV, a leucocidin, inhibited HCC cell migration by downregulating HDAC6, thus enhancing α-tubulin acetylation in a concentration-dependent manner [93]. AR420626, a selective agonist of G-protein coupled receptor GPR41/FFA3, suppressed the growth of HCC cells by inducing apoptosis thereby reducing the expression of HDAC2-7 with an increase in histone H3 acetylation [94]. High expression of HDAC8 was observed in both tumor tissues and liver cancer cell lines while its suppression effectively disrupted cancer cell dynamics by reducing cell proliferation and triggering programmed cell death [95]. Moreover, Yang and colleagues highlighted that HDAC8 inhibition can increase T cell infiltration into tumors and decrease regulatory T cells (Tregs), thus improving the immune response against HCC [96]. An in vivo study reported a positive correlation between HDAC9 and programmed death-ligand 1 (PD-L1) expression levels. Moreover, the combination of high levels of HDAC9 and of PD-L1 was associated with a decreasing overall survival (OS) in patients with HCC [97]. HDAC11 deacetylates Egr-1, consequently suppressing the expression of p53 and promoting liver cancer progression [98] (Figure 3). Nevertheless, studies investigating the role of HDAC11 in liver cancer are limited.

Current research has highlighted the profound significance of epigenetic regulation in cancer pathogenesis, particularly liver cancer. Epigenetic alterations have displayed a unique therapeutic opportunity, offering a promising avenue for developing novel strategies that can potentially interrupt or reverse cancer development at the molecular level [99]. Therefore, altered expression of HDACs could play an active role in tumor onset and progression and make them attractive candidate targets for anticancer drugs and therapies.

## 5. Therapeutic Implications: Promising Synthetic HDAC Inhibitors in HCC

HDACs are among the most promising therapeutic targets for cancer treatment. HDAC inhibitors are the compounds targeting epigenetic dysregulation—a hallmark of cancer. HDAC inhibitors have direct anti-tumor effects, modulating the tumor microenvironment. Therefore, HDAC inhibitors have been targeted for the development of anticancer strategies as well as therapies for human diseases [100,101,102]. The utilization of multiple therapeutic agents in the treatment of cancer is another useful anticancer strategy [103,104]. Currently, clinical trials are demonstrating the effectiveness of HDAC inhibitors (HDACis) alone and in combination with other drugs [105,106]. A few HDAC inhibitors have been approved by the Food and Drug Administration (FDA) for the treatment of different types of cancer [107]. Although, at present, no HDACi is approved for the treatment of HCC, some are currently the object of clinical or preclinical trials for HCC treatment. In particular Resminostat and several HDACis, when used in combination with other treatment modalities, have also demonstrated enhanced therapeutic efficacy against HCC [108,109,110]. Treatment with HDAC inhibitors was shown to upregulate the expression of checkpoint inhibitors in tumor cells. A selective HDAC1/2/3 inhibitor (CXD101) has overcome resistance to immune checkpoint therapy in liver cancer by activating an IFNγ/STAT1 signaling and GSDME-mediated pyroptosis circuit, effectively converting immune-excluded tumors into inflamed ones that respond better to treatment. This finding has progressed to a phase-II clinical trial and could provide a new treatment strategy for patients with immunotherapy-resistant liver cancer [111]. HDAC inhibitors have also been investigated in combination with oncolytic virotherapy to promote tumor immunogenicity in hepatocellular carcinoma [112,113].

Another emerging field of research focuses on nicotinamide N-methyltransferase (NNMT), a NAD regulator, that influences the activity of sirtuins, NAD-dependent histone deacetylases. NNMT could be the target of specific inhibitors that can be used in liver cancer treatment [114,115]. These inhibitors function through multiple mechanisms including immunomodulation, apoptosis induction and cell cycle arrest with current clinical trials testing both monotherapy and combination approaches with standard chemotherapy, immunotherapy and targeted agents [116,117].

In the following paragraphs, we will illustrate the recent advances found in the literature regarding selected synthetic HDACis with, a focus on those targeting zinc-dependent HDACs, highlighting their antitumoral function and clinical relevance related to HCC treatment. Table 2 illustrates the most important HDACis with related characteristics.

### 5.1. Resminostat

Resminostat is an oral, potent bioavailable pan-inhibitor of class I and II HDACs. It is a member of benzenes, a hydroxamic acid, a member of pyrroles, a sulfonamide, a tertiary amino compound, and an enamide. Resminostat binds to and inhibits HDACs leading to an accumulation of highly acetylated histones. This results in the induction of chromatin remodeling, transcription of tumor suppressor genes, tumor cell apoptosis, and inhibition of tumor cell division. Resminostat has shown particular promise in addressing the epigenetic dysregulation common in HCC development and has been used in clinical trials studying the treatment of many cancers [133,134,135]. Clinical studies with Resminostat in HCC have produced encouraging results, particularly in combination with Sorafenib. Its development program has specifically focused on HCC patients, with trials examining its potential in both first line and second-line settings, making it one of the most thoroughly studied HDAC in the context of liver cancer. In earlier studies, Resminostat showed additive or synergistic activities in combination with other novel pharmaceutical agents and conventional chemotherapeutic agents for HCC treatment [136]. The SHELTER study where Resminostat was investigated as a second-line therapy for advanced HCC reported that combination of Resminostat with Sorafenib improved progression-free survival (PFS) to 6.5 months and overall survival (OS) to 8.0 months as compared to 1.8 months PFS and 4.1 months OS with Resminostat alone, suggesting its potential in overcoming therapy resistance and exhibiting promising clinical outcomes in terms of safety and survival [118]. Resminostat also exhibited potent anti-HCC activity by activating the mitochondrial permeability transition pore (mPTP) dependent apoptosis pathway, which led to cytochrome C release and caspase-9 activation in HCC cell lines and primary HCC cells. When combined with Sorafenib, Resminostat synergistically enhanced the anti-tumor effects through amplified activation of the mitochondrial apoptosis pathway [119]. In another study, Resminostat was found to stimulate mesenchymal HCC cells to have a more epithelial phenotype with lower invasive activity, downregulating CD44 (a cancer stem cell marker) that may contribute to the sensitization to Sorafenib-induced apoptosis, preventing HCC cells growth in vitro [120]. It is worth noting that in a Phase I/II trial comparing first-line Sorafenib plus Resminostat versus Sorafenib alone in East Asian patients with advanced HCC, no significant improvement in overall survival with the combination therapy was found. However exploratory analyses suggested potential benefits in patients with normal to high baseline platelet counts or HBV-related HCC [137]. Previously, researchers demonstrated that inhibiting mTOR significantly enhanced the anticancer effects of Resminostat in hepatocellular carcinoma cells, with the combination activating the mitochondrial apoptosis pathway more potently. This finding revealed that mTOR activation may be a key resistance mechanism against HDAC inhibitor therapy in liver cancer, suggesting that combining HDACis with mTOR inhibitors could be a promising therapeutic strategy [138].

### 5.2. Vorinostat

Vorinostat is a histone deacetylase inhibitor of class I and II, a hydroxamic acid, and a dicarboxylic acid diamide. It has a role as an apoptosis inducer and an antineoplastic agent. It binds to the zinc atom in the catalytic domain of HDAC enzymes and projects its phenyl ring out of the catalytic site onto the surface of HDAC enzymes. Thus, HDACs cannot remove the acetyl group of histone and non-histone proteins, provoking the accumulation of acetylated proteins with effects in many cellular functions, such as cell cycle arrest in cancer cells. It has been approved by FDA for the treatment of cutaneous T-cell lymphoma [139]. Vorinostat, also known as suberoylanilide hydroxamic acid (SAHA), effectively overcomes Lenvatinib resistance in HCC by suppressing the PI3K/AKT signaling pathway, revealing that AKT activation contributes to Lenvatinib resistance, while HDAC inhibition restores sensitivity to treatment. Both in vitro and in vivo findings support the potential of Vorinostat, and Lenvatinib combination therapy, as a promising strategy to enhance therapeutic efficacy and counteract drug resistance in HCC [121]. Vorinostat combined with the DNMT inhibitor Decitabine has also shown effective results in solid tumors, including HCC. It has synergistically enhanced the antitumor effects in HCC cells by inducing apoptosis and autophagy mediated by caspase-3 activation, Bcl-2 downregulation, and autophagic flux upregulation [122]. Studies demonstrating Vorinostat ability to induce apoptosis and prevent cell growth in HCC cell lines include that by Li and colleagues [123] who reported that Vorinostat triggers apoptosis through caspase activation and downregulation of anti-apoptotic proteins, while Sanaei and colleagues [140] showed its role in inhibiting HCC cell proliferation by modulating key oncogenic pathways.

### 5.3. Panobinostat

Panobinostat is a hydroxamic acid, a member of cinnamamides, a secondary amino compound, and a methylindole. It acts as an HDACi, an antineoplastic agent, and an angiogenesis-modulating agent, and it has been approved by the FDA in 2015. It is used for the treatment of relapsed/refractory (R/R) multiple myeloma [141], and recently it has been used to also treat brain cancer [142]. Some studies have reported Panobinostat’s promising anti-HCC efficacy both in vitro and in vivo with minimal adverse effects. Liu and colleagues [124] have reported that Panobinostat treatment, particularly when combined with NAT10 (a histone acetyltransferase) silencing, significantly reduced HCC cell lung metastasis as well as noticeably decreased liver tumor growth in xenograft and lung metastasis mouse models. Panobinostat also showed enhanced anti-tumor effect in HCC cells when combined with radiotherapy [143]. Some studies report the enhanced induction of apoptosis as well as proton sensitization when treated with Panobinostat in HCC cells [125,126] in addition to autophagy-induced cell death [144]. Panobinostat treatment led to the silencing of 5 out of 6 miRNA of the oncogenic miR17-92 cluster in HCC cell lines ultimately reducing carcinogenesis [145].

### 5.4. Romidepsin

Romidepsin is a cyclodepsipeptide classified as an organic disulfide and a heterocyclic antibiotic, which has been approved by FDA in the treatment of cutaneous T-cell lymphoma and peripheral T-cell lymphoma [146]. The possible effectiveness of Romidepsin in the treatment of HCC is still under investigation but preliminary studies have yielded promising results. In vitro studies demonstrate that Romidepsin inhibits cell cycle and induces apoptosis [127,147] in HCC cell lines. These findings were corroborated in tumor xenograft mouse models where Romidepsin effectively suppressed HCC cell growth. Further studies on diethylnitrosamine (DEN)-induced HCC mouse models have shown that Romidepsin treatment led to a significant suppression of hepatocellular tumorigenesis [128,148]. A retrospective study demonstrated the pro-apoptotic effects of Romidepsin in HCC cells by mediating G2/M arrest through the activation of JNK/MAPK and Erk/MAPK pathways as shown in an in vivo mouse model and in an in vitro drug sensitivity assay [149]. A recent study on the combination therapy approach, involving the co-administration of Romidepsin with PARP inhibitor Olaparib, showed an efficient decrease in HCC growth both in vivo and in vitro [129].

### 5.5. Belinostat

Belinostat is a hydroxamic acid, a sulfonamide, and an olefinic compound with antineoplastic activity. In 2014, it has been approved by the FDA, and it is widely used to treat several cancers such as T-cell lymphoma, soft tissue sarcoma, mesothelioma, colorectal cancer, B-cell lymphoma, liver cancer and refractory or relapsed PTCL [139]. Belinostat in combination with another drug, Bortezomib, has shown antiproliferative and cytotoxicity properties towards HCC cell lines [130]. Moreover, a previous study also demonstrated that Belinostat inhibits growth and induces apoptosis in HCC cell lines with the partial restoration in expression of some silenced tumor suppressor genes [131]. Belinostat has also shown to enhance antitumor immunity when combined with the immune checkpoint inhibitor anti-CTL4 in murine HCC models [132]. Belinostat also showed enhanced cytotoxicity against HCC cell lines even in over-expressed ornithine decarboxylase 1 (ODC1) HCC cells [150].

## 6. Natural Dietary Components as HDAC Inhibitors

**Figure 4:**
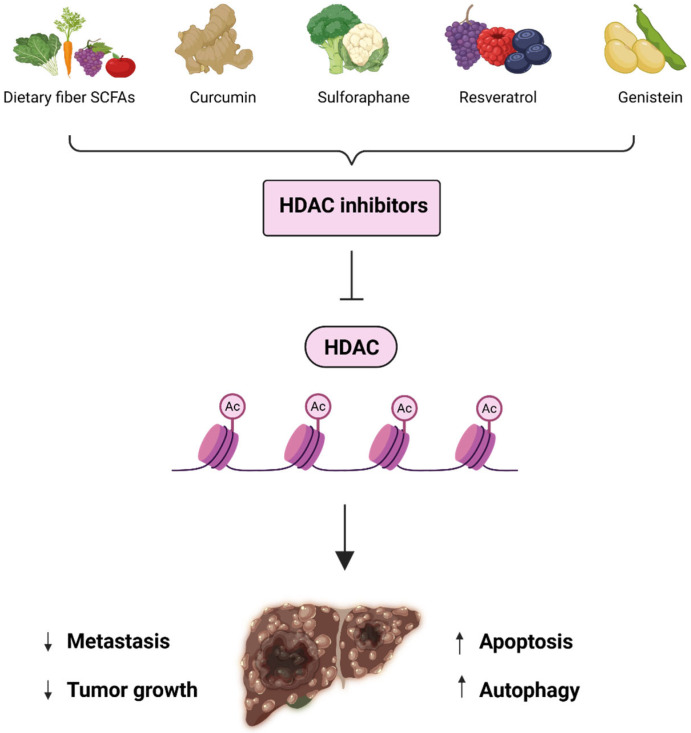
Role of natural HDAC inhibitors in Hepatocellular carcinoma. Compounds from gut microbiome and dietary sources act as natural histone deacetylase inhibitors by augmenting acetylation and halting tumorigenesis in HCC.

Natural compounds from various dietary and plant sources can serve as effective histone deacetylase inhibitors (Figure 4), providing a novel avenue for chemoprevention and therapy [151,152,153,154]. These bioactive compounds show both selective and multiple target actions against HDAC enzymes while maintaining low toxic effects (Table 3). Intersection of nutrition and epigenetics ascertains that several dietary components function as molecular switches, controlling gene expression patterns in different pathologies including cancer.

### 6.1. Short-Chain Fatty Acids (SCFAs)

Short-chain fatty acids are produced by gut microbiota through the fermentation of dietary fibers. SCFAs are organic acids with carbon chain containing less than six carbon atoms. Acetate, butyrate, propionate and valeric acid are the most common SCFAs. They act as an endogenous HDAC inhibitors [155]. Acetate suppressed IL-17A production by type 3 innate lymphoid cells through the inhibitory activity on HDAC and induction of Sox13 (SRY-box transcription factor 13) acetylation in a dose-dependent manner. Acetate administration with PD-1 therapy delayed tumor growth and improved anticancer immunity in the HCC mouse model [156]. Propionate upregulated activation of caspase-3 and cisplatin-induced apoptosis through a TNF-α-induced pathway. Augmented acetylation of H3 by reducing the expression of HDACs via GPCR activation is also reported. Sodium propionate in combination with cisplatin significantly increased apoptosis and suppressed growth of HCC xenografts [157]. A high concentration of sodium butyrate (NaB) in sorafenib-resistant HCC cells showed reduced cell viability and increased cell apoptosis as well as the decreased expression of miRNA-7641 and miRNA-199 [158]. Butyrate in combination with chloroquine exerted cytotoxic activity in HCC tumors and increased the production of reactive oxygen species. Variation in the levels of autophagy markers are also reported [159]. The NaB and DNMT inhibitor indicated inhibition in cell growth and induction in apoptosis while upregulating the expression of some TSGs and apoptotic regulators [160]. The HDAC inhibitor NaB upregulated the expression of tumor suppressor gene *Cyld* in HCC cells through enhanced histone acetylation [161]. Increased expression of sarco-endoplasmic reticulum Ca^2+^-ATPase 3 (SERCA3) is detected through NaB and TSA treatment in HCC cells with an increase in H3 acetylation on the respective *SERCA3* gene [162]. Butyrate, selectively, induces apoptosis to cancerous cells while supporting normal cells, with effects varying according to cell type and dose. It is evident that at high concentrations (2mM), butyrate exhibited apoptosis of both cancerous and non-cancerous colonocytes. Low availability has limited its clinical use but strategies like nanodelivery, structural analogs, coupling with phytochemicals, and serine conjugation can improve its therapeutic potential [163,164]. Valeric acid (VA) has shown significant anticancer effects including reduced colony formation, migration, and invasion of liver cancer cells. This study also investigated the role of encapsulated valeric acid for liver cancer cells in xenograft mouse models, indicating efficient results. Both VA and encapsulated VA showed reduced HDAC activity as well [165]. As short-chain fatty acids have notable anticancer and anti-inflammatory properties and improve physiological function, studies are reported regarding SCFA oral supplementation as a therapeutic strategy. However, several factors can reduce the bioavailability of SCFAs over time, due to their potential decline in the gut, such as aging, metabolic syndrome, and cardiovascular diseases [166,167,168]. 

### 6.2. Curcumin

Curcumin is a polyphenolic compound, naturally occurring in turmeric spice, that also acts as a natural HDAC inhibitor. Curcumin exerted its tumor-initiating cell (TIC) depleting activity partially through inhibiting NF-kB signaling. Curcumin-sensitive HCC cells showed reduced stemness and tumorigenicity by downregulating HDAC class I and II [169]. Curcumin and Trichostatin A can reduce cell growth and induce apoptosis in HCC cells. Curcumin showed dose- and time-dependent substantial antiproliferative effects, more significant apoptotic effect than TSA and the upregulation of *ERα* gene expression [170]. The bioavailability of curcumin is compromised, due to its rapid metabolism and its low serum and tissue levels. Studies are being conducted to enhance its bioavailability using delivery approaches including nanoparticles, liposomes, or its derivatives. Curcumin has shown no harmful consequences at high dosage. Curcuminoids are FDA-recognized as safe and well-tolerated in clinical trials at doses of 4000–8000 mg [171,172].

### 6.3. Sulforaphane

Sulforaphane is an isothiocyanate found mostly in cruciferous vegetables and functions as a dietary HDACi. Sulforaphane significantly downregulated the expression of *HDAC5* and *HDAC11* leading to the upregulation of several genes involved in DNA damage response and cell cycle. Sulforaphane also determined reduced cell viability and induced apoptosis in HCC cells and it possibly regulates oncogenic transcription factors via methylation of their DNA binding motifs [173]. In acute liver failure mouse models, sulforaphane has been shown to reduce HDAC6 expression by inhibiting ferroptosis through activation of NRF2 [174]. Since NRF2 plays a central role in triggering the antioxidant response in various cell types, this mechanism is particularly relevant given the key involvement of oxidative stress regulation in the ferroptosis process [175,176,177,178].

Systemic availability of sulforaphane is highly dependent on the form of administration as well as the presence and activity of myrosinase, an enzyme for plant defense. Fresh broccoli sprouts retain active myrosinase and are the most effective dietary source of sulforaphane [179,180]. No adverse side effect has been observed with the high dose of sulforaphane (50 μM) on HCC cells except mild reversible effects exhibiting minimal toxicity [181].

### 6.4. Resveratrol

Resveratrol is a polyphenol abundant in grapes and berries, and it acts as an HDAC suppressing compound. Resveratrol suppressed all eleven human HDACs, from HDAC1 to 11 in a dose-dependent manner in an in vitro analysis and showed antiproliferative effects on HCC cell lines. Interestingly, Resveratrol showed significant HDAC inhibition and consequently histone hyperacetylation in HepG2 cells. Further testing on human blood detected a resveratrol-mediated inhibition of HDAC in PBMCs, demonstrating its high concentration in blood can modulate HDAC activity [182]. Nowrasteh and colleagues evaluated the effect of nutrient rich fruit extract including trans-resveratrol in cancer induced mice models and demonstrated the significant reduction in expression of *HDAC2*, *HDAC3* and a few *DNMTs* [183]. Although resveratrol possesses several beneficial biological activities [184], it has low bioavailability and solubility. A dose of 100–500 mg is recommended for its supplementation to avoid potential risks. Resveratrol bioavailability enhancement of oral administration when in combination with other compounds is under investigation [185,186].

### 6.5. Genistein

Genistein is an isoflavone, a type of flavonoid and a natural HDACi present in soy products and legumes. It has shown apoptotic and antiproliferative effects alone and in combination with TSA on HCC cell lines. In addition, it also directed the re-expression of silenced gene estrogen receptor alpha *ERα* in the HCC cell line [187]. Sanaei and colleagues had also shown the restoration of gene expression of some DNA methyltransferases through Genistein and TSA in addition to cell growth inhibition and apoptosis induction. They also studied the effects of Genistein alone and in combination with Valproic acid [188,189]. According to the US-FDA, daily consumption of 25 g of soy containing genistein is recommended without side effects. Despite showing promising anticancer effects [190,191], pharmacokinetic analysis of genistein has shown low oral bioavailability, which is a major hurdle in its application in clinical studies [192].

**Table 3:** Common natural HDAC inhibitors studied in HCC models.

Compound and Structure	Molecular Formula (MW)	Source	Study Model	Key Findings	Ref.
Acetic acid 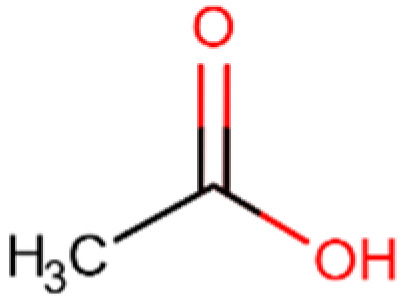	C_2_H_4_O_2_ (60.05 g/mol)	Natural SCFA; Bifidobacteria	In vivo and ex vivo	Anti-tumor activity, efficient immunity	[156]
Propionic acid 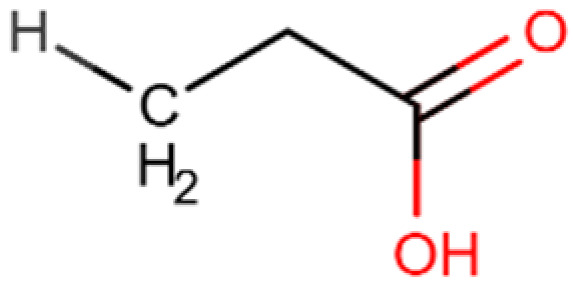	C_3_H_6_O_2_ (74.08 g/mol)	Natural SCFA; Bacteroidetes	Cell lines; Xenograft mouse model	Tumor regression, enhanced apoptosis and H3 acetylation	[157]
Butyric acid 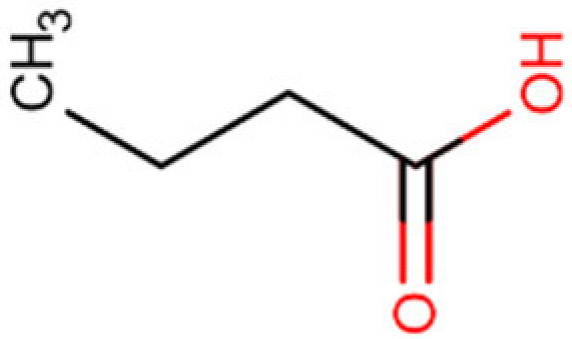	C_4_H_8_O_2_ (88.11 g/mol)	Natural SCFA; Firmicutes	Cell lines	Cytotoxic activity, increased acetylation	[159,161,162]
Valeric acid 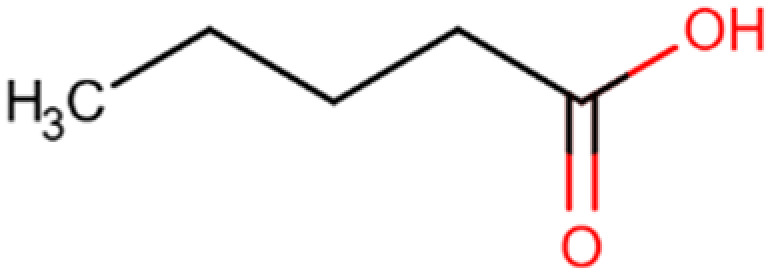	C_5_H_10_O_2_ (102.13 g/mol)	Natural SCFA; Clostridia	Cell lines; Xenograft mouse model	Antitumor effects	[165]
Curcumin 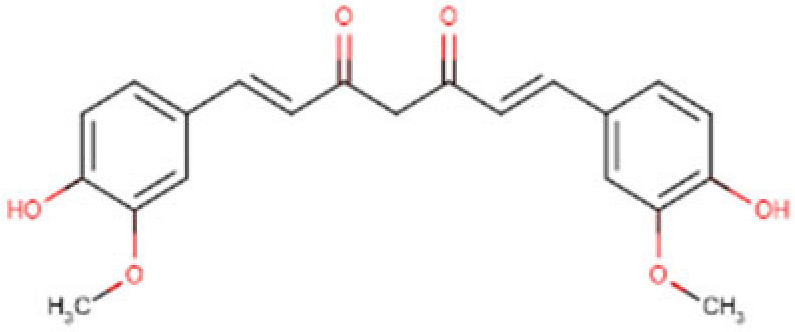	C_21_H_20_O_6_ (368.4 g/mol)	Turmeric (*Curcuma longa*)	Cell lines	Apoptotic and antiproliferative activity	[169,170]
Sulforaphane 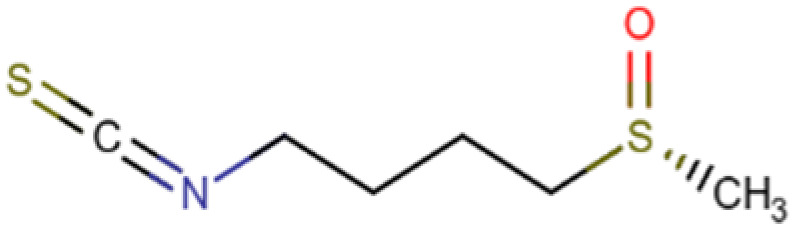	C_6_H_11_NOS_2_ (177.3 g/mol)	Cruciferous vegetables	Cell lines	Apoptosis induction, gene regulation	[173]
Resveratrol 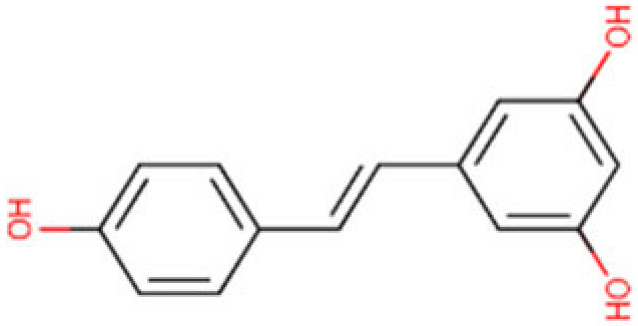	C_14_H_12_O_3_ (228.24 g/mol)	Red grapes	In vitro and ex vivo	Antiproliferative effects	[182]
Genistein 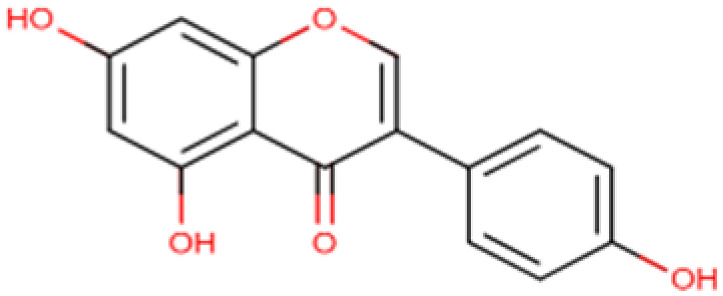	C_15_H_10_O_5_ (270.24 g/mol)	Soy products	Cell lines	Cell proliferation inhibition, increase apoptosis	[187,188]

## 7. Future Perspectives and Conclusions

Recent advances in the development of novel epigenetic-targeted drugs demonstrated significant potential in preclinical and clinical trials [26,27]. These therapeutic approaches aim to modulate aberrant epigenetic modifications and restore physiological cellular function, offering a promising frontier in cancer treatment.

Despite the plethora of studies reported in this review, some questions remain uncovered and are still matters of debate such as the cytoplasmic effects of HDACi, the interplay between their anti-apoptotic and autophagic effects, overall effect on epigenetic crosstalk during treatment, etc. Another critical issue is related to non-histone targets of HDACs, since HDAC inhibition can trigger widespread downstream effects, limiting therapeutic specificity. Moreover, recently, a study reported that radiotherapy can impart a long-term epigenetic memory in skin cells. It is an open and critical question whether tumor cells similarly retain an epigenetic memory of treatment. This memory could be detrimental or potentially advantageous and its implications are completely unknown [193]. Regarding a more general perspective, while the goal is often “epigenome normalization,” it is still not proven whether this is the inhibitors’ main mechanism of action due to their pleiotropic nature.

Nevertheless, several challenges remain in optimizing HDAC inhibitor therapy regardless of promising results. Studies on natural HDAC inhibitors are predominantly focused on in vivo and in vitro experimentations, lacking robust and randomized trials in humans. Their heterogenous mechanism of action, low bioavailability, rapid metabolism, and short half-lives interfere with their efficacy and applicability. Conversely, synthetic HDAC inhibitors are extensively evaluated in clinical studies but face several challenges like isoform selectivity, pharmacokinetics and epigenetic plasticity. Furthermore, limited efficacy as a monotherapy, off-target interactions, and disparity in clinical trial design are other aspects of their limited therapeutic capability. Another major limitation in the real-life use of HDACs inhibitors refers to the limited knowledge in defining the most appropriate dosage as well as timing, meaning the stadium of disease, in which HDACs could reach the most beneficial effects.

Current research should focus on developing more selective and natural HDACis, identifying reliable biomarkers for patient stratification and optimizing combination strategies. The emergence of precision medicine approaches, coupled with advanced molecular profiling techniques, is enabling more personalized therapeutic strategies, potentially leading to improved clinical outcomes for hepatocellular carcinoma patients. The future of HDAC-targeted therapy likely lies in rational combination strategies incorporating HDACis with conventional chemotherapeutics, immunotherapies, oncolytic viral therapy and other epigenetic regulators. Translational research should also explore next-generation HDACis with enhanced selectivity and reduced off-target effects ensuring better clinical applicability. Healthy lifestyle and nutrient rich diet based preventive approaches could also contribute to epigenetic resilience and reduced cancer risk. Characterization of the cellular and molecular mechanisms by which specific epigenomic reprogramming elicits anti-tumor immunity is instrumental in the rational development and clinical translation of selective HDAC-targeted immunotherapy. Conclusively, HDAC inhibition remains a promising therapeutic avenue for hepatocellular carcinoma. However, maximizing clinical efficacy demands further elucidation of mechanisms of resistance, patient stratification and identification of synergistic drug combinations. Given the complexity of the epigenetic network and sheer number of potential targets, a systematic approach identifying and validating potential drug targets is imperative to focus on drug development and confirm the potential of this strategy.

## Figures and Tables

**Table 1:** HDACs characteristics based on data from Human Protein Atlas.

Protein	Gene ID	Aliases	Chr	HDAC Class	Cellular Localization	Histone Substrates	Non-Histone Substrates
HDAC1	3065	GON-10, HD1, KDAC1, RPD3, RPD3L1	1p35.2	I	Nucleus, Cytoplasm	Lysine residues of H2A, H2B, H3, H4	NR1D2, RELA, SP1, SP3, STAT3 and TSHZ3
HDAC2	3066	HD2, KDAC2, RPD3, YAF1	6q21	I	Nucleus, Cytoplasm	Lysine residues of H2A, H2B, H3, H4	TSHZ3
HDAC3	8841	HD3, KDAC3, RPD3, RPD3-2	5q31.3	I	Plasma membrane, Nucleus, Cytoplasm	H3K27	KAT5, MEF2D, MAPK14, STAT3 and RARA
HDAC4	9759	AHO3, BDMR, HA6116, HD4, HDAC-4, HDAC-A, HDACA, NEDCHF, NEDCHID	2q37.3	IIa	Nucleus, Cytoplasm	Lysine residues of H2A, H2B, H3, H4	HSPA1A and HSPA1B at Lys-77
HDAC5	10014	HD5, NY-CO-9	17q21.31	IIa	Nucleus, Cytoplasm	Lysine residues of H2A, H2B, H3, H4	RARA
HDAC6	10013	CPBHM, HD6, JM21, KDAC6, PPP1R90	Xp11.23	IIb	Plasma membrane, Nucleus, Cytoplasm	Lysine residues of H2A, H2B, H3, H4	Tubulin, α-tubulin, SQSTM1 and CTTN
HDAC7	51564	HD7, HD7AA, HDAC7	12q13.11	IIa	Nucleus, Cytoplasm	Lysine residues of H2A, H2B, H3, H4	RARA and ALKBH5
HDAC8	55869	CDA07, CDLS5, HD8, HDACL1, KDAC8, MRXS6, RPD3, WTS	Xq13.1	I	Plasma membrane, Nucleus, Cytoplasm	Lysine residues of H2A, H2B, H3, H4	SMC3
HDAC9	9734	ARCND4, HD7, HD7b, HD9, HDAC, HDAC7, HDAC7BB, HDAC9FL, HDRP, MITR	7p21.1	IIa	Nucleus, Cytoplasm	Lysine residues of H2A, H2B, H3, H4	-
HDAC10	83933	HD10	22q13.33	IIb	Nucleus, Cytoplasm	Lysine residues of H2A, H2B, H3, H4	MSH2
HDAC11	79885	HD11	3p25.1	IV	Plasma membrane, Nucleus, Cytoplasm	Lysine residues of H2A, H2B, H3, H4	-

NR1D2, Nuclear Receptor Subfamily 1 Group D Member 2; RELA, V-Rel Avian Reticuloendotheliosis Viral Oncogene Homolog A; SP1, specificity protein 1; SP3, specificity protein 3; STAT, signal transducers and activator of transcription 3; TSHZ3, Teashirt Zinc Finger Homeobox 3; KAT5, Lysine Acetyltransferase 5; MEF2D, Myocyte Enhancer Factor 2D; MAPK14, Mitogen-Activated Protein Kinase 14; RARA, Retinoic Acid Receptor Alpha; HSPA1A, Heat Shock Protein Family A (Hsp70) Member 1; HSPA1B, Heat Shock Protein Family A (Hsp70) Member 1B; SQSTM1, Sequestosome 1; CTTN, Cortactin; ALKBH5, AlkB Homolog 5; SMC3, Structural Maintenance Of Chromosomes 3; MSH2, MutS Homolog 2.

**Table 2:** Comprehensive Profile of Synthetic HDAC Inhibitors in HCC models.

HDAC Inhibitor Name (Trade Name) and Structure	Aliases	Molecular Formula (MW)	Study Design/Model	Key Findings	Ref.
Resminostat (Kinselby) 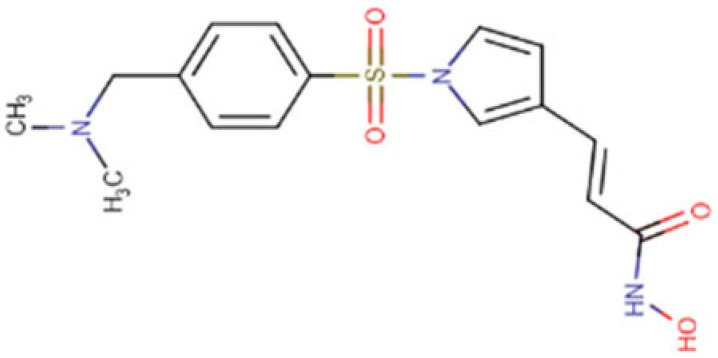	4SC-201 RAS2410 BYK408740	C_16_H_19_N_3_O_4_S (349.4 g/mol)	Phase I/II clinical trial; in vitro studies	Apoptosis induction; enhanced antitumor activity; improved efficacy with Sorafenib	[118,119,120]
Vorinostat (Zolinza) 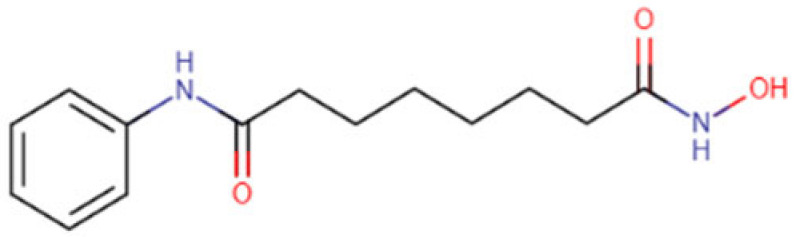	SAHA MK0683	C_14_H_20_N_2_O_3_ (264.32 g/mol)	In vitro and in vivo studies	Improved efficiency with Lenvatinib; synergistic anticancer effect via dual induction of apoptosis and autophagy	[121,122,123]
Panobinostat (Farydak) 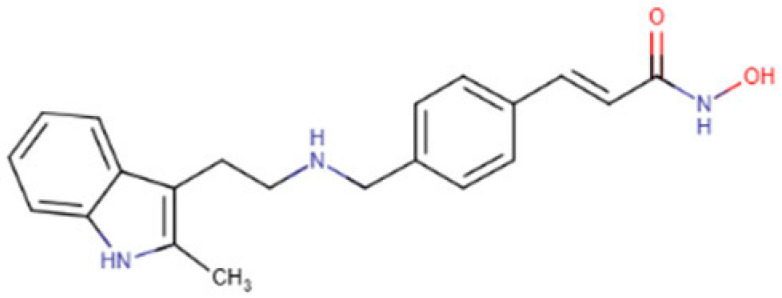	LBH589	C_21_H_23_N_3_O_2_ (349.4 g/mol)	Xenograft mouse models; cell lines	Reduction in lung metastasis; enhanced antitumor effect and apoptosis induction in combination with radiotherapy	[124,125,126]
Romidepsin (Istodax) 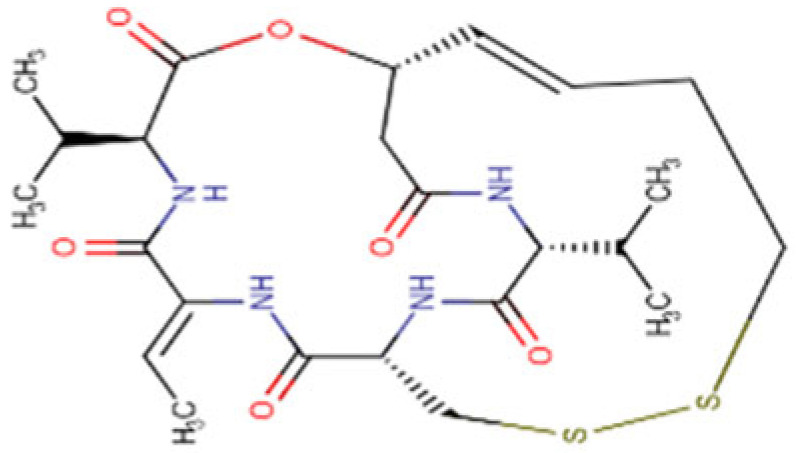	Depsipeptide Chromadax FK228	C_24_H_36_N_4_O_6_S_2_ (540.7 g/mol)	In vitro and in vivo studies	Cell cycle arrest; HCC tumor suppression; enhanced outcome with Olaparib	[127,128,129]
Belinostat (Beleodaq) 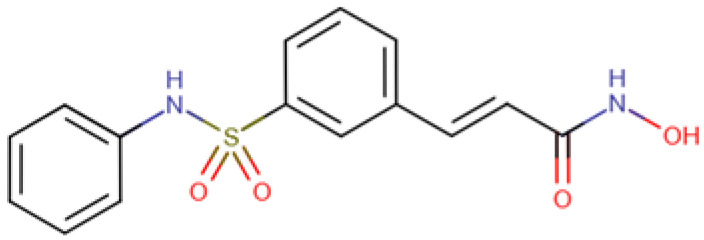	PXD101	C_15_H_14_N_2_O_4_S (318.3 g/mol)	Cell lines; murine models	Antiproliferative and cytotoxic effects with Bortezomib; apoptotic induction and cell suppression; enhanced anti-tumor immunity	[130,131,132]

## Data Availability

No new data were created or analyzed in this study. Data sharing is not applicable to this article.

## Abbreviations

The following abbreviations are used in this manuscript:

CoREST	Corepressor of REST
DEN	Diethynitrosamine
EMT	Epithelial–mesenchymal transition
HATs	Histone acetyltransferases
HCC	Hepatocellular carcinomas
HIF1α	Hypoxia-inducible factor 1 α
HDACi	Histone deacetylase inhibitor
HDAC	Histone deacetylase
MAD	Matrix associated deacetylase
MEF2	Myocyte enhancer factor 2
mPTP	Mitochondrial permeability transition pore
N-CoR	Nuclear receptor co-repressor
NaB	Sodium butyrate
ncRNA	non-coding RNA
NES	Nuclear export signal
NLS	Nuclear localization signal
NuRD	Nucleosome remodeling and deacetylase complex
ODC1	Ornithine decarboxylase 1
OS	Overall survival
PD-L1	Programmed death ligand 1
PFS	Progression free survival
PTM	Post-translational modification
Rpd3	Reduced potassium dependency protein 3
SAHA	Suberoylanilide hydroxamic acid
SCFA	Short-chain fatty acid
Sir2	Silent information regulator 2
Treg	Regulatory T cell
TSA	Trichostatin A
TSG	Tumor suppressor gene
VA	Valeric acid
ZnF	Zinc finger domain
